# The multistep path to replicative senescence onset: zooming on triggering and inhibitory events at telomeric DNA

**DOI:** 10.3389/fcell.2023.1250264

**Published:** 2023-09-13

**Authors:** Paolo Pizzul, Carlo Rinaldi, Diego Bonetti

**Affiliations:** Dipartimento di Biotecnologie e Bioscienze, Università di Milano-Bicocca, Milan, Italy

**Keywords:** telomere, senescence, cancer, homologous recombination, yeast, R-loops

## Abstract

Replicative senescence is an essential cellular process playing important physiological functions, but it is better known for its implications in aging, cancer, and other pathologies. One of the main triggers of replicative senescence is telomere shortening and/or its dysfunction and, therefore, a deep understanding of the molecular determinants is crucial. However, replicative senescence is a heterogeneous and hard to study process, especially in mammalian cells, and some important questions still need an answer. These questions concern i) the exact molecular causes triggering replicative senescence, ii) the role of DNA repair mechanisms and iii) the importance of R-loops at telomeres in regulating senescence onset, and iv) the mechanisms underlying the bypass of replicative senescence. In this review, we will report and discuss recent findings about these mechanisms both in mammalian cells and in the model organism *Saccharomyces cerevisiae*.

## 1 Introduction

The ends of eukaryotic linear chromosomes must be protected to ensure genome stability and cell survival. Specialized nucleoprotein complexes, called telomeres, carry out these crucial functions. Telomeres consist of short tandem DNA repeats (e.g., TTAGGG in mammals) and of a group of specialized proteins, known as “shelterin” in mammals, which regulate telomere functions (reviewed in de Lange, 2018; Casari et al., 2022).

Telomere length is critical because it is directly proportional to the protective capacity. Telomeres become progressively shorter upon each cell division due to the “end replication problem” and nucleolytic degradation (Levy et al., 1992; Fallet et al., 2014; Sholes et al., 2022). When telomeres become critically short and/or telomeric proteins are lost, chromosome ends become “uncapped” and they are recognized as DNA damage (reviewed in de Lange, 2018; Galli et al., 2021).

Telomere shortening can be counteracted by two telomere maintenance mechanisms (TMMs): i) a specific enzyme, called telomerase, that synthesizes new telomeric DNA in a tightly controlled manner; ii) homology-directed DNA repair mechanisms (HDR) collectively called “Alternative Lengthening of Telomeres” (ALT). However, in most human cells, telomere shortening is not sufficiently counteracted and robust TMM activation is a feature of cancer cells.

Telomeres have important implications in cancer and aging because they are involved in a process called “replicative senescence” that is tightly linked in these two cellular events. Replicative senescence is a permanent proliferation arrest triggered by several events, including telomere shortening and/or deprotection. It has long been considered as a tumor suppressor mechanism because it limits the proliferation of cells with oncogenic mutations and it prevents genome instability caused by excessive telomere shortening and deprotection (reviewed in Maciejowski and de Lange, 2017; Casari et al., 2022). Recent findings, however, highlight a positive role for replicative senescence in cancer evolution, related to the secretory functions of senescent cells (Yang et al., 2021). Last but not least, the accumulation of senescent cells can deteriorate tissue functions, thereby linking replicative senescence to aging and age-related diseases, which include cancer (reviewed in Hernandez-Segura et al., 2018).

Cellular senescence is a double-edged sword and this evidence emphasizes the importance of its deeper understanding both to unravel pathological process and to find new therapeutic approaches.

Here, we will review some recent findings concerning the connections between telomeres and replicative senescence both in mammals and in the model organism *Saccharomyces cerevisiae*.

## 2 Replicative senescence onset and its regulation

### 2.1 Telomeres and replicative senescence

Telomere shortening is not sufficiently counteracted in most human cells and this physiological event contributes to limit cell proliferation (Allsopp et al., 1992). Indeed, critically shortened and/or dysfunctional telomeres elicit a DNA damage response (DDR), which triggers cell death or an irreversible arrest in cell division, the latter known as “replicative senescence” state or the “M1 stage” (IJpma and Greider, 2003; d’Adda di Fagagna et al., 2003; Galli et al., 2021). It is noteworthy that mitochondrial defects, non-telomeric DNA damage, chromatin changes, and oncogenes activation also contribute to replicative senescence onset (Gorgoulis et al., 2019).

The exact mechanisms through which telomeres trigger replicative senescence onset are still not fully understood. In yeast cells, a single very short telomere is sufficient to trigger replicative senescence, while in mammalian cells multiple (from 5 to 10) very short telomeres are required (Zou et al., 2004; Kaul et al., 2011; Xu et al., 2013). However, the exact threshold length and, more importantly, the exact state of the shortest telomere(s) triggering replicative senescence are still unclear. Moreover, i) how short or damaged telomeres activate and maintain the DDR, ii) whether and how cells decide to repair them, and iii) how DNA repair contributes to replicative senescence onset are still open questions.

**FIGURE 1:**
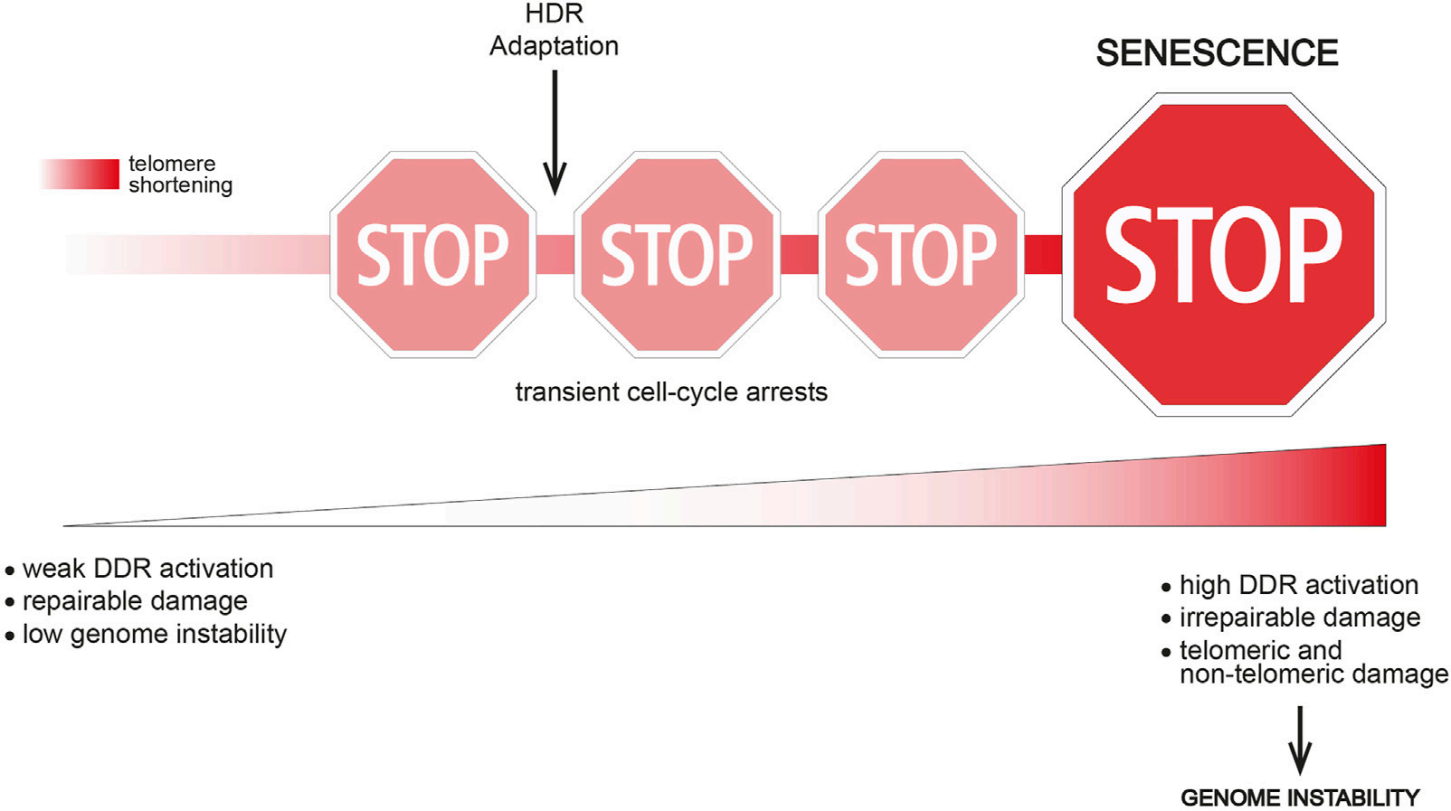
The route to replicative senescence. Replicative senescence onset is a multi-step process in which transient cell-cycle arrests occur before the terminal arrest. Transient arrests might be caused by the presence of a repairable damage (e.g., partial telomere uncapping, stochastic DNA damage) or replication stress at telomeres. A weak and confined DDR response might be another possible cause of a transient arrest. Genome instability is likely still low at this point. Cell division arrests might be overcome i) by DNA repair activities (e.g., HDR), eventually leading to telomere elongation, ii) by tolerating a weak DDR response or iii) by switching off the DDR and trying to survive without solving the DNA damage (adaptation). Adaptation clearly leads to increased genome instability, while HDR contribution to genome instability is not clear. The event(s) triggering the terminal senescent arrest might be critically short/irreparable telomere(s), as well as a broader DNA damage. In both cases, an excessive genome instability and/or DDR activation overcoming a certain threshold lead to replicative senescence onset.

Interestingly, single-cell studies in the model organism *S*. *cerevisiae* have shown that telomerase-negative yeast cells often undergo transient periods of cell division arrest followed by resumption of normal growth before finally entering a “terminal” state (replicative senescence) (Xu et al., 2015; Coutelier et al., 2018). Importantly, “non-terminal” arrests start to occur early after telomerase inactivation, when telomere shortening is not yet critical, thus indicating that this event is not the triggering signal. However, the frequency of transient arrests increases with ongoing cells divisions and telomere shortening, thus suggesting that short/dysfunctional telomeres contribute to reach the terminal arrest (Figure 1). Interestingly, transient proliferation arrests preceding replicative senescence have been described in human cells too (Ghadaouia et al., 2018; Ghadaouia et al., 2021).

These transient arrests indicate the presence of DNA damage and DDR activation. Several hypotheses could explain how cells resume growth: telomeric damage i) is resolved, ii) activates a weak DDR that is tolerated, iii) remains unrepaired and the DDR is switched off in a process called “adaptation” (Figure 1). These findings also suggest that different signals reflecting different telomere length/state exist and they dictate whether the arrest is transient or permanent.

The exact trigger of the terminal arrest is still unclear, especially in mammalian cells. Here, multiple short/dysfunctional telomeres might be required to pass a DDR activation threshold.

Interestingly, genome instability increases upon subsequent non-terminal arrests both in yeast and in mammals (Coutelier et al., 2018; Ghadaouia et al., 2018; Ghadaouia et al., 2021). An intriguing hypothesis suggests that dysfunctional telomeres are not direct inducers of replicative senescence, but they rather lead to a wider genome instability and a strong DDR activation that together trigger senescence onset (Figure 1). Accordingly, adaptation to DNA damage is a potent source of genome instability (Pizzul et al., 2022), which may accumulate upon successive transient arrests. In human cells, telomere uncapping can be tolerated leading to a transient arrest (Ghadaouia et al., 2018; Ghadaouia et al., 2021), but subsequent cell divisions with dysfunctional telomeres likely fuel genome instability and lead to a stable senescence-associated proliferation arrest (Ghadaouia et al., 2021).

### 2.2 HDR and replicative senescence

The path to replicative senescence onset must be tightly regulated to ensure a non-pathogenic balance. In fact, the premature accumulation of senescent cells contributes to progeroid syndromes (reviewed in Armanios, 2022). As previously mentioned, replicative senescence affects both cancer suppression and development. For example, solid tumors are rare in individuals with short telomere syndromes associated with premature senescence, whereas long telomeres seem to promote cancer development, likely by postponing senescence onset and increasing the chance for oncogenic mutations to occur (McNally et al., 2019; Armanios, 2022). Thus, since telomere length and protection are critical determinants for senescence onset, their control is essential.

Telomeres constitute a barrier against DNA repair mechanisms. Nonetheless, in yeast and mammals, homology-directed DNA repair (HDR) mechanisms constitute a TMM besides telomerase enzyme (Lundblad and Blackburn, 1993; Bryan et al., 1997; Sobinoff and Pickett, 2017). Telomeres can be lengthened by the recombination-mediated synthesis using the DNA sequences of other telomeres as a template. HDR is important for telomere maintenance in a subset of cancer cells (ALT cells) and in yeast cells (called “survivors”) that bypass replicative senescence and upregulate/deregulate these mechanisms at telomeres (Lundblad and Blackburn, 1993; Le et al., 1999; Apte and Cooper, 2016).

Importantly, HDR is also crucial before senescence onset, but these mechanisms are still poorly understood, especially in mammals. In yeast cells, HDR impairment in the absence of telomerase leads to a drastically premature senescence (Claussin and Chang, 2016). Notably, in telomerase-negative cells, many important HDR factors bind telomeres long before senescence onset (Khadaroo et al., 2009; Pérez- Martínez et al., 2020). Recently, RAD51 defects have been shown to cause premature aging in mouse models (Matos-Rodrigues et al., 2022). HDR activity was also shown to decrease as human fibroblasts get closer to replicative senescence (Mao et al., 2012). Thus, under certain circumstances, recognition of a telomere as a DNA damage is not triggering senescence but rather contributes to its physiological delay.

The exact role of HDR mechanisms in regulating replicative senescence onset is still unclear. Since HDR can elongate telomeres, this event may be directly important in delaying senescence onset (Kockler et al., 2021). By telomere sequencing, rare telomere addition events can be detected in telomerase-negative yeast cells before senescence onset (Claussin and Chang, 2016; Sholes et al., 2022). Moreover, recent nanopore sequencing approaches have shown that, in telomerase-negative yeast cells, subpopulations with sets of elongated telomeres are drastically reduced in the absence of HDR (Sholes et al., 2022), in contrast with previous findings (Claussin and Chang, 2016).

**FIGURE 2:**
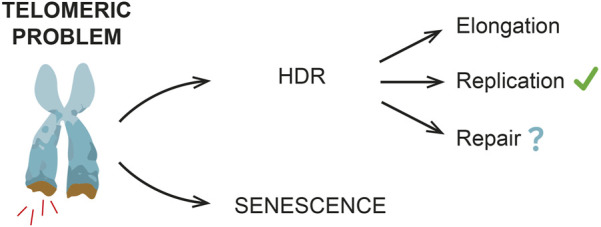
HDR and replicative senescent onset. HDR mechanisms contribute to delay a premature senescence onset, even if in mammals this evidence is not as clear as in yeast. Different HDR roles have been reported, including telomere elongation and resolution of DNA replication stress. Other DNA repair events are also possible, especially during early non-terminal arrests, when telomeres have not reached a critical length yet (e.g., stochastic telomeric DNA damage).

Alternatively, HDR might be required to protect/resolve telomeres under replication stress (Claussin and Chang, 2016; Matos-Rodrigues et al., 2022) and/or limit the signal(s) triggering senescence onset, with no telomere elongation (Figure 2).

As previously mentioned, both in yeast and in human cells, transient cell division arrests have been observed, thus suggesting the existence of a “reversible” telomeric damage. Here is where HDR likely acts (Figure 2), thus preventing premature and frequent terminal arrests. In yeast, indeed, the lack of HDR machinery strongly reduces the frequency of transient arrests (Xu et al., 2015).

Nonetheless, an important question is still open: is HDR a safe process that helps to avoid a premature senescence or is it a backup mechanism that lays the groundwork for senescence onset? A recent work by Kockler and others proposed that, in yeast cells, shortened telomeres undergo HDR events that prevent a premature senescence onset by elongating telomeres, but, at the same time, as cells undergo subsequent cell divisions and arrests, genome instability increases. Accordingly, recent findings in human cells suggest that HDR might occur at uncapped telomeres and these events contribute to increase genome instability and promote senescence onset (Ghadaouia et al., 2018; Ghadaouia et al., 2021).

Altogether these data suggest that HDR delays premature senescence onset, but at the same time it might promote genome instability and set the starting point for senescence onset. Further studies are required to better understand these processes.

### 2.3 R-loops and replicative senescence

Important mechanisms of HDR regulation at telomeres rely on R-loop formation. R-loop is a three-stranded nucleic acid structure where the formation of a RNA-DNA hybrid, mainly during transcription, displaces the second DNA strand. R-loops are receiving huge attention because they play important biological functions but, on the other hand, their dysregulation contributes to genome instability and to the development of different diseases [reviewed in García-Muse and Aguilera (2019) and in Rinaldi et al. (2021)].

R-loops form in many genomic regions, including telomeres. Here, their formation is mainly ascribed to the transcription of TERRA, a long non-coding RNA conserved from yeast to mammals that plays important roles in regulating both TMMs (reviewed in Fernandes et al., 2021; Zeinoun et al., 2023). It is still debated whether R-loops form only *in cis* during TERRA transcription or even *in trans* when the transcribed TERRA anneals back to the telomere (Feretzaki et al., 2020). Nonetheless, these structures clearly play important roles in the regulation of HDR at telomeres, both before senescence onset and in ALT cells.

Indeed, several studies in yeast and mammals have shown that TERRA and R-loops levels are high both in pre-senescent and in ALT cells, and they are important for telomere maintenance by HDR (Arora et al., 2014; Graf et al., 2017; Misino et al., 2018; Guh et al., 2022; Misino et al., 2022). Moreover, they regulate telomere length dynamics and senescence onset in pre-senescent yeast cells by promoting HDR: accumulation of R-loops delays senescent onset and this effect requires HDR, while an increased removal of RNA-DNA hybrids at telomeres accelerates senescence onset (Balk et al., 2013; Graf et al., 2017).

How exactly R-loops promote HDR is still unclear. The most likely hypothesis is that, as in other genomic regions, R-loops hamper DNA replication, leading to replication stress and the formation of DNA double-strand breaks (DSBs) that are repaired by HDR. R-loops may also act by recruiting repair factors to telomeres and promote HDR initiation (D’Alessandro et al., 2018). TERRA has recently been shown to interact with RAD51, a key player in HDR, and RAD51 is important for TERRA R-loop formation at telomeres (Feretzaki et al., 2020). Thus, it is also possible that increased levels of TERRA and R-loops may increase the local concentration of RAD51 to sustain DNA recombination. Accordingly, TERRA has been recently shown to interact with proteins involved in several DNA repair pathways (Guh et al., 2022).

Several studies have shown that R-loops must achieve an optimal balance to stimulate HDR. In fact, their excess causes an overload of replication stress or hampers the DNA repair machinery access, thus destroying telomere integrity (Arora et al., 2014; Balk et al., 2014; Silva et al., 2021).

These findings suggest that telomere maintenance via HDR requires several factors regulating R-loop levels, both positively and negatively. Indeed, new factors are continuously identified or shown to be involved at telomeres in addition to other genomic regions (Silva et al., 2019; Guh et al., 2022; Kaminski et al., 2022; Pérez-Martínez et al., 2022).

In this scenario, the role of important factors, like RNA-DNA and RNA helicases, still need to be investigated at telomeres. Indeed, in yeast, two important helicases involved in R-loop regulation, Sen1 and Pif1, orthologs of mammalian SETX and PIF1, have been shown to interact with telomere specifically during replicative senescence (Pérez-Martínez et al., 2022). Many RNA helicases, especially those belonging to the DEAD-box family, have been identified as telomere interactors and their role need to be deeply addressed (Pérez-Martínez et al., 2022).

## 3 Yeast as a model organism

The budding yeast *S. cerevisiae* took the stage as a tool for the study of telomere biology very early in the history (reviewed in Casari et al., 2022). Thanks to the high evolutive conservation of telomere biology, it quickly became a model organism for understanding the mechanisms of i) telomere maintenance, ii) replicative senescence onset, and iii) replicative senescence bypass.

Beyond its easy manipulation and the powerful genetic engineering tools, *S*. *cerevisiae* offers many advantages to study telomere biology: i) it expresses telomerase enzyme, ii) it rapidly reaches the senescent state upon telomerase depletion, iii) cells that overcome senescence and maintain telomeres by HDR, called “survivors”, easily arise.

Clearly, the use of yeast as a model has some limitations, including i) the lower complexity of telomere structure (e.g., mammalian t-loops) and of proteins networks controlling telomere protection compared to mammals; ii) a simplified HDR machinery and a different balance in NHEJ vs. HDR DNA repair mechanisms compared to mammals, and iii) the knowledge that in multicellular organisms there has been an evolutive selection for replicative senescence (Kowald et al., 2020).

In the study of replicative senescence, yeast offers the interesting and unique opportunity to set a “time zero” by genetically inactivating telomerase, thus allowing to follow the senescence process from the beginning in a nearly synchronous way. This approach also offers important opportunities to compare different genotypes and to elaborate the senescence kinetics. This is usually done by deriving haploid telomerase-negative cells from a heterozygous diploid or by controlling telomerase expression through regulatable promoters. Upon telomerase depletion, yeast telomeres shorten up to 5 bp per generation (Sholes et al., 2022). At the onset of senescence, around 60–80 population doublings after telomerase depletion, the average telomere length in the population is 100–120 bp but the shortest telomere is about 75 bp long (Kockler et al., 2021; Sholes et al., 2022).

Yeast is also a very important tool to study ALT mechanisms. Indeed, many ALT features are conserved from yeast to human cells. About 15% of cancers activate ALT as a TMM (Apte and Cooper, 2016), which is also responsible for relapses of cancers treated with anti-telomerase drugs (Hu et al., 2012). The significant role that these mechanisms play in human diseases makes it important to understand how ALT is initiated, established, and maintained. However, current human cell models mainly allow the study of ALT maintenance, whereas its establishment remains obscure. The use of *S. cerevisiae* offers the opportunity to address mechanisms underlying the bypass of replicative senescence arrest and the establishment of cells that can proliferate by activating ALT mechanisms.

A recent important upgrade in this scenario has been introduced by Kockler and others. Before the development of their new methods, post-senescence yeast survivor formation was mainly a “yes” or “no” answer and the study of cell populations did not allow to catch and analyze single events. Now the possibility to study the frequency is significantly increasing our understanding of ALT mechanisms.

Another recent intriguing discovery is that also yeast survivors experience replicative senescence. Indeed, by propagating cells starting from single clones after survivor formation, Misino and others observed that these cells undergo cycles of senescence, similarly to non-survivors. They refer to this event as “survivor associated senescence” (SAS), highlighting an unexpected parallel between senescent and post-senescent cells (Misino et al., 2022).

## 4 Conclusion and perspectives

Since replicative senescence is tightly linked to aging and cancer, future studies are essential to understand some pathological processes and to develop new therapeutic strategies. In fact, as recently shown, a deeper and more complete understanding of senescence can be useful to figure out its complex role in the evolution of cancer and to identify new therapeutic targets based on different types of senescent cells (Garbarino et al., 2023).

The current “state of the art” is that replicative senescence onset is not a straightforward process, in which at some point one or more critically short telomeres trigger a permanent DDR to prevent genome instability. Rather, it appears to be a multistep process in which many aspects still need to be clarified. Importantly, further studies are required to clarify the exact signal(s) that triggers senescence and especially the contribution of genome instability in both senescent onset and bypass. It is important to better understand the role of HDR during replicative senescence onset and what are exactly the outcomes at telomeres that avoid a premature senescence rather than promoting it. Moreover, it is important to elucidate the role of R-loops and their regulation at telomeres during replicative senescence onset and in ALT cells.

Several omics approaches will be useful, both in yeast and mammals, to better understand many of these molecular mechanisms and to find an answer to the different open questions. Yeast, for example, is a powerful tool to perform genetic screenings, and recent genome editing technologies will allow to perform genome-wide genetic screenings also in human cells. In particular, the identification of specific alleles could be particularly useful when knock-outs are not possible or too drastic, as for several HDR genes. Different interesting approaches to scan for specific mutations in specific genes have been successfully applied in human cells (Kweon et al., 2020; Cuella-Martin et al., 2021).

Similarly to yeast, single-cell analyses are a powerful tool to be applied to mammalian cells to better understand replicative senescence (Cohn et al., 2023; Fard et al., 2023). Since to date, in mammals, replicative senescence has been mainly studied at the bulk-population level and results depend on cells source, age, telomerase expression, and many other factors. Moreover, cultures of senescent cells consist of a mix of cell types, including proliferative and apoptotic cells. Therefore, new technologies are required to identify and characterize senescent cells with more accuracy.

A combination of genetic and genomics approaches together with a better identification of senescent cells during time course analyses will help to clarify several open questions, including the role of HDR mechanisms during replicative senescence.
